# Microplastics and neurotoxicity: could prenatal exposure to microplastics boost congenital enteric neuropathies?

**DOI:** 10.3389/ftox.2026.1756622

**Published:** 2026-03-24

**Authors:** Rasul Khasanov, Michael Boettcher, Lucas M. Wessel, Karl-Herbert Schäfer, María Ángeles Tapia-Laliena

**Affiliations:** 1 Department of Pediatric Surgery, Medical Faculty of Mannheim, University of Heidelberg, Mannheim, Germany; 2 Working Group Enteric Nervous Systems (AGENS), University of Applied Sciences Kaiserslautern, Campus Zweibrücken, Kaiserslautern, Germany

**Keywords:** congenital enteric neuropathies, enteric nervous system (ENS), Hirschsprung’s disease (HSCR), microplastics, neurotoxicity, pregnancy

## Abstract

Microplastics (MPs) pollution represents an increasing worldwide problem and a real global challenge for human health, which also affects unborn children. Specifically, during their degradation, they can release a broad range of toxic and hormonally active agents, such as plasticizers. Thus, microplastics alone are pernicious, but they often also carry other harmful chemicals and even problematic bacteria on their surface and within their structure (heavy metals, pesticides, parabens, etc.), which amplifies their toxic potential. Due to their induction of oxidative damage, inflammation, mitochondrial apoptosis, and microbiota dysbiosis, and more, microplastics act as neurotoxic agents. Periods particularly sensitive to this neurotoxicity include fetal development and childhood, during which microplastics can negatively affect proper neuronal development. When expecting mothers are exposed, microplastics can cross the placenta barrier, reach the developing embryo, and accumulate in its organs. During fetal development, even minor interferences in neuronal migration can result in deficient neuronal innervation in the gut, potentially leading to congenital enteric neuropathy. Although an accurate estimation of human exposure is still pending, this may produce serious intestinal motility disorders and compromise the long-term quality of life of newborns. In this review, we analyze how microplastic neurotoxicity could be an aggravating factor in the development of congenital enteric aganglionosis and, consequently, postnatal motility disorders. Finally, we propose reducing pregnant women’s exposure to microplastics as an important preventive measure to protect the fetus from neurotoxicity.

## 1 Introduction

### 1.1 Microplastic exposure: an emerging health problem

During the last decades, global plastic production has grown exponentially (Geyer et al., 2017), resulting in plastic waste reaching all ecosystems (Geyer et al., 2017; Senathirajah et al., 2021). As these plastics reach the end of their life cycle, they degrade under the effects of mechanical erosion, sunlight, air, or water (Julienne et al., 2019) into smaller pieces, classified by size into microplastics (MPs, ranging from 5 mm to 0.1 μm) or nanoplastics (NPs, <0.1 μm). Among these degraded fragments, the most abundant polymer types are polypropylene (PP), polyethylene (PE), polyethylene terephthalate (PET), polystyrene (PS), polycarbonate (PC), poly-(methyl methacrylate) (PMMA), and polyvinyl chloride (PVC) (Dubey et al., 2022; Nair et al., 2025). Furthermore, additional plasticizers, such as diethylhexyl-phthalate (DEHP), which are released during the degradation of PVC, have also demonstrated their considerable toxic potential, particularly for the liver (Loff et al., 2000).

Consequently, MPs have, over the years, reached and polluted most ecosystems worldwide (Dubey et al., 2022) and have already been detected ubiquitously in water, soil, and air (Lu et al., 2025; Nair et al., 2025). Furthermore, MPs have emerged as a significant and pervasive health and environmental problem globally (Campanale et al., 2020) as they are persistent, toxic, and possess bioaccumulative potential (Weis and Alava, 2023).

Industry, paints, and tire wear, and sewage sludge (Kwiatkowska and Ormaniec, 2024; Thompson et al., 2024) contribute significantly to MPs emissions into the environment (Kole et al., 2017; Paruta et al., 2022). However, MPs can also be found in a wide array of products, including plastic packaging, synthetic textiles (Rovira and Domingo, 2019), seafood (Ferrante et al., 2022; Vega-Herrera et al., 2024), fruits and vegetables (Oliveri Conti et al., 2020), salt, sugar, tap and bottled water (Panneerselvam et al., 2025), and even cosmetics and personal care products (Guerranti et al., 2019; Sun et al., 2020; Cubas et al., 2022). They also enter the food chain almost inevitably, originating from food packaging, cooking pots, and plastic bottles (Gelbke et al., 2019; van Raamsdonk et al., 2020). Hence, MPs abundance in the food chain has already been proven by several studies (Cox et al., 2019; Senathirajah et al., 2021; Gambino et al., 2022). Due to their accumulation in the food chain, MPs disrupt the delicate balance of ecosystems and may impair cognitive function and behavioral patterns in living organisms (Lu et al., 2025). As a result, humans are continuously exposed to microplastic-induced toxicity from the environment on a daily basis (Nair et al., 2025).

MPs can enter the human body through various ways, including ingestion, inhalation, and dermal contact with personal care products (Cox et al., 2019; Lu et al., 2025; Panneerselvam et al., 2025). Truly, we are breathing (Baeza-Martínez et al., 2022), eating, and drinking them every day, so they later accumulate in our intestines (Zhu et al., 2024), tissues, and organs (Deng et al., 2017; Gelbke et al., 2019; van Raamsdonk et al., 2020; Nihart et al., 2025).

Once they enter the body, MPs show a systematic distribution (Bhattacharyya et al., 2025), being detected in all human samples, except for kidneys and cerebrospinal fluid (Bai et al., 2024). They have also been isolated in the human respiratory system (Baeza-Martínez et al., 2022) and in human stool (Schwabl et al., 2019). MPs can penetrate physiological barriers in humans, such as the placental, blood–brain, and blood–testis barriers (Bai et al., 2024; Bhattacharyya et al., 2025; Lu et al., 2025). This barrier transfer depends on MPs characteristics, exposure dose, route, time, co-exposure to other pollutants, and genetic predisposition.

Chronic exposure to MPs has been associated with several organ-specific toxicities, including gastrointestinal toxicity, hepatotoxicity, neurotoxicity, reproductive and developmental toxicities (Dubey et al., 2022; Bai et al., 2024), and, more recently, with carcinogenesis (Gutiérrez-García et al., 2025).

Finally, maternal exposure to MPs has been reported to cause adverse effects on pregnancy outcomes in pregnant mice (Hu et al., 2021) and is related to reproductive toxicity and low growth offspring, also in humans (Luo et al., 2019).

However, an accurate estimation of human exposure is still pending due to the lack of standardized methods for processing and analyzing MPs in human samples (Catalán et al., 2025). For instance, most of the research was conducted using mainly polystyrene (PS), but this polymer represents only 7% of total plastic production. In addition, differently sized particles, concentrations, incubation times, and models have been analyzed, complicating the later comparison of results (Brachner et al., 2020).

Recently, further controversy has arisen regarding the accuracy of MPs quantification methods, calling into question previous MPs studies on both the environment (Evangelou et al., 2026) and human tissues (Monikh et al., 2025) and consequently sparking a renewed debate on the challenges of achieving precise methodologies (Brachner et al., 2020; Thompson et al., 2024; Monikh et al., 2025; Campen et al., 2025). Nevertheless, efforts are underway to unify protocols and improve methodology in the future (Altmann et al., 2025; Catalán et al., 2025; Nardella et al., 2025).

### 1.2 Microplastics induce neurotoxicity

Although the neurotoxic effects of MPs vary according to individual particle types, shapes, sizes, exposure concentrations, and durations, it is clear that exposure to MPs implicates a neurotoxic risk (see Table 1) (Prüst et al., 2020).

**TABLE 1:** Overview of MPs neurotoxicity mechanisms.

Mechanism	References	Associated with	References
↑Oxidative stress	Cubas et al. (2022) and Ullah et al. (2023)	Neurodegeneration	Lu et al. (2025)
Endocrine disruption	Bai et al. (2024) and Panneerselvam et al. (2025)	Neurodevelopmental issues	Deng et al. (2017), Freire et al. (2018), and Singh et al. (2022)
Microbiota dysbiosis	Fournier et al. (2023), Lee et al. (2024), and Saraluck et al. (2024)	Behavioral	Prüst et al. (2020)
Inflammation	Bhattacharyya et al. (2025)	Dementia	Bhattacharyya et al. (2025) and Lu et al. (2025)
Inhibition of acetylcholinesterase	Prüst et al. (2020)		
Neurotransmitter disruption	Prüst et al. (2020)	Cognitive	Deng et al. (2017); Jin et al. (2018), (2019); and Zheng et al. (2024)
↑Autophagia	Lu et al. (2025)		
Mitochondrial disruption	Prüst et al. (2020)	Behavioral	Zheng et al. (2024)

Ingested MPs can disrupt gut microbiota, breach intestinal and blood–brain barriers, and accumulate in neural tissues through systemic distribution (Bhattacharyya et al., 2025). Specifically, the disruption of the mucous barrier might present a gateway for bacterial metabolites and toxins, thus adding to the direct effect of the toxic microplastic components a secondary by-stander effect, which severely aggravates their impact.

Mechanistic studies have revealed that MPs induce oxidative stress, leading to cellular damage, neuroinflammation, protein aggregation, alterations in neurotransmitter levels, and inhibition of acetylcholinesterase activity (Prüst et al., 2020). MPs exposure has also been related to mitochondrial injury, inflammatory signaling, and impaired protein homeostasis (Prüst et al., 2020; Bhattacharyya et al., 2025). Given this evidence, MPs have been shown to induce neurotoxicity, brain damage, and impaired neuronal development in different animal models (Pahwa and Kalra, 1993; Qu and Wang, 2020; Sökmen et al., 2020; Zheng et al., 2024).

Moreover, ingested MPs appear to increase neurological risk and contribute to the development of cognitive dysfunction and pathways leading to neurodegenerative diseases. Not surprisingly, high MPs burdens in brain tissue have been associated with dementia, neuronal disorders (Bhattacharyya et al., 2025; Nihart et al., 2025), behavioral alterations, and increasing rates of neurodegenerative diseases (Lu et al., 2025).

On top of that, MPs contain other toxic chemicals within their structure but also adsorb additional toxicants from the environment on their surfaces (Brachner et al., 2020), including polychlorinated biphenyls (PCBs), pesticides, heavy metals (Al, Cd, Co, Cr, Cu, Hg, Mn, and Pb), polycyclic aromatic hydrocarbons (PAHs), persistent organic pollutants (POPs), and antibiotics (Cubas et al., 2022; Weis and Alava, 2023; Rafa et al., 2024). Some of these contaminants are commonly used as additives in plastic production. As they are not covalently bonded to plastics, they can easily leach into milk, water, and other liquids (Ullah et al., 2023). MPs particles act as solid substrates for various microorganisms, promoting the formation of microbial biofilms with different metabolic activities (Kwiatkowska and Ormaniec, 2024).

As concerning evidence, MPs particles have been detected to leach from medical parenteral nutrition bags used in neonatal intensive care into the administered nutritional solution, from where they may reach the digestive system of the premature parenterally fed neonates but may also cause obstruction and pulmonary inflammation since they exceeded the diameter of lung and tissue capillaries (Vercauteren et al., 2024). Previous studies associated parenteral nutrition in premature neonates with serious hepatobiliary dysfunction (Loff et al., 1998; 1999). Previously, parenteral nutrition was assumed to be responsible for liver damage. Now, MPs leaching into parenteral nutrition circuits have emerged as a plausible cause.

The physicochemical properties of microplastics, such as size, structure, and functional groups, and properties of environmental compartments, such as pH, temperature, and salinity, influence the sorption of pollutants by microplastics (Rafa et al., 2024). Toxicity induced by MPs and NPs is size-dependent as smaller particles have better absorption capacity and larger surface area, releasing more toxic chemicals and endocrine disruptors, leading to oxidative stress, reproductive toxicity, neurotoxicity, cytotoxicity, developmental abnormalities, decreased sperm quality, and immunotoxicity (Ullah et al., 2023).

All these toxic pollutants transported by MPs can bioaccumulate and induce oxidative stress and cellular toxicity (Cubas et al., 2022). For instance, phthalates have been linked to various neurotoxic mechanisms, such as endocrine disruption, oxidative stress, and cellular apoptosis (Tseng et al., 2013; Wójtowicz et al., 2017).

Human exposure to metals has also been shown to induce neurotoxicity through various mechanisms such as autophagy (Zhang et al., 2013), synaptic transmission (Braga et al., 1999), oxidative stress (Chew et al., 2011), and alterations in neurotransmitter metabolism (Pohanka, 2014). Regarding metals, even low concentrations of As, Pb, Cd, and Hg during gestation must be considered due to their severe effects on the fetus. In particular, Cd is harmful because when Cd accumulates in the placental tissue, other metals such as As, Cr, Cu, Pb, Mn, Ni, Se, and Zn are, as a consequence, transferred from maternal tissue to the placenta and from there to the fetus, putting the newborn’s life at risk (Serafim et al., 2012) and particularly affecting neurodevelopment (Meyrueix et al., 2019).

Other common additives in MPs, including phthalates, bisphenol A, and parabens (Iribarne-Durán et al., 2022; Mejías et al., 2023), are particularly concerning due to their interference with the neuroendocrine system, which plays a crucial role in regulating physiological functions. Furthermore, studies indicate that when MPs are present alongside these harmful additives, they may exhibit synergistic toxicity, which intensifies the detrimental effects (Brachner et al., 2020; Lu et al., 2025).

Thus, MPs can not only trigger neurotoxicity and inflammation but also enhance endocrine disruption (Bai et al., 2024; Kim et al., 2025). Regarding this, we should not forget that pregnancy is a particularly vulnerable period for the potential risks of endocrine disruptors (Leppert et al., 2020).

### 1.3 Microplastics can cross the placental barrier and accumulate in fetal tissues

MPs have been detected in a variety of human tissues, including ovarian follicular fluid (Montano et al., 2025), maternal blood and urine (Sun et al., 2024), the placenta (Bissonnette et al., 2025; Nair et al., 2025), and even the umbilical cord (see Table 2).

**TABLE 2:** Detection of microplastics and their co-adsorbed pollutants in maternal and fetal samples.

Sample	Pollutant	References
Ovarian follicular fluid	Microplastics	Montano et al. (2025)
Placenta	Microplastics	Aengenheister et al. (2018), Campanale et al. (2020), Braun et al. (2021), Ragusa et al. (2021), Bissonnette et al. (2025), Kim et al. (2025), and Nair et al. (2025)
	Metals	Freire et al. (2018)
	Parabens	Vela-Soria et al. (2017)
	Benzophenones	Vela-Soria et al. (2017)
Ammniotic fluid	Microplastics	Sun et al. (2024)
Umbilical cord	Microplastics	Sun et al. (2024)
	Benzophenones	DiNardo and Downs (2019)
Maternal urine/blood	Microplastics	DiNardo and Downs (2019) and Sun et al. (2024)
	Benzophenones	Huo et al. (2016) and DiNardo and Downs (2019)
	Parabens	Leppert et al. (2020)
Breast milk	Microplastics	Liu et al. (2023) and Saraluck et al. (2024)
	Bisphenol	Iribarne-Durán et al. (2022)
	Parabens	Iribarne-Durán et al. (2022) and Zhang et al. (2023)
	Benzophenones	Iribarne-Durán et al. (2022)
Meconium	Microplastics	Braun et al. (2021) and Liu et al. (2023)
Infant feces	Microplastics	Liu et al. (2023)

MPs have been found in maternal and infant paired samples (Bai et al., 2024), indicating that MPs can cross the placenta and blood–brain barrier, reaching fetal tissues, where they accumulate (Bai et al., 2024; Kim et al., 2025). In addition, other pollutants adsorbed to MPs, such as metals (Freire et al., 2019), parabens, and ultraviolet filters (Vela-Soria et al., 2017), can cross the placental barrier. The detection of MPs in the first neonate feces, known as meconium, provides further evidence that MPs can reach the fetus (Braun et al., 2021; Liu et al., 2023).

In animal experiments, when MPs derived from disposable paper cups (DPCs) were administered to pregnant mice, the particles were later detected in multiple tissues, with preferential accumulation in the fetus, placenta, kidney, spleen, lung, and heart, contributing to impaired phenotypes. A dose-responsive harmful effect on fetal development and maternal physiology was observed (Chen et al., 2024).

Exposure of mated mice to polystyrene nanoplastics (PS-MPs) showed an elevated embryo resorption rate during the peri-implantation period that led to a reduction in the number and diameter of uterine arterioles, which might reduce the uterine blood supply and increase miscarriage risk. In addition, disturbances in immune cell and cytokine secretion were observed (Hu et al., 2021).

Prenatal exposure to toxins may be especially harmful, given that the developing brain is a more susceptible target to toxicity as neurons proliferate, migrate, and forge important synaptic connections to create an optimum adult brain structure (Rodier, 1995).

Animal experiments have demonstrated that exposure to PS-NPs influences the development of the offspring’s hippocampus and induces neurotoxicity, which contributes to a reduction in learning and memory function (Tian et al., 2025). Another study linked maternal exposure to MPs, such as polystyrene, to the development of metabolic disorders in the offspring (Luo et al., 2019).

Recent research has found a higher number of MPs (mainly PET and PPC) in the feces of infants than in adults, indicating that infants are exposed to higher levels of MPs than adults and to a probable microplastic accumulation during the gestation period (Zhang et al., 2021).

Worryingly, exposure to these pollutants continues after pregnancy: evidence shows the presence and concentrations of MPs (Liu et al., 2023; Saraluck et al., 2024), bisphenols, parabens (PBs), and benzophenones (BPs) in human breast milk (Iribarne-Durán et al., 2022) as well as in infant formula (Liu et al., 2023) and processed infant food products, such as milk-based infant formula and cereal-based complementary foods (Zhang et al., 2023). This evidence indicates that babies are exposed even after the gestational period, as also demonstrated by later MPs identification in infant feces (Liu et al., 2023).

In addition, MPs have been observed to change the bacterial composition of human milk (Saraluck et al., 2024), which may have implications for the establishment of the newborn’s intestinal microbiota. As another piece of evidence, a study using *in vitro* infant gut models has already found that MPs ingestion induces perturbations in the intestinal microbiome (Fournier et al., 2023).

In short, MPs found in blood, breast milk, and placenta raise concerns about fetal development and long-term health, compromising endocrine function and neurological development issues in offspring (Panneerselvam et al., 2025). Currently, a European project (AURORA) is underway to assess the effects of MPs exposure during pregnancy and early life (Durkin et al., 2024).

## 2 Microplastic neurotoxicity may disrupt enteric neuronal migration

The pregnancy period is highly sensitive, such that disturbances at any sequential steps can alter the normal embryonic development, during which a single-celled zygote undergoes multiple divisions and differentiations to give rise to a fully formed multicellular organism.

Exposure to MPs and NPs during early developmental stages may trigger neurotoxicity and inflammatory responses, which could cause abnormal embryonic development (Lu et al., 2025; Nair et al., 2025).

This is especially true for congenital enteric neuropathies, such as Hirschsprung disease (HSCR, incidence 1/5,000), which is characterized by a deficient gastrointestinal innervation of the colon due to a failure of enteric neural crest cell migration during early embryogenesis (from 5 to 12 weeks) (Heuckeroth, 2018). The regulation of this process is critical, with numerous genes and proteins involved in both migratory and colonization events (Lake and Heuckeroth, 2013). Although some mutations have been identified in HSCR, the condition is not fully explained by the genetic load (Tang et al., 2018; Luzón-Toro et al., 2020), and many non-genetic factors could also impair the development of the enteric nervous system (ENS) and contribute to the risk (Heuckeroth, 2018). Therefore, identifying an environmental trigger may help prevent HSCR in some cases (Heuckeroth, 2015).

The main treatment for HSCR involves the surgical removal of the deficiently innervated (aganglionic) segment of the intestine. Later on, many patients undergo complications such as recalcitrant constipation, toxic megacolon, and enterocolitis, leading to compromised quality of life (Menezes and Puri, 2006). On the other hand, minor impacts on ENS development may not be sufficient to cause HSCR but may still produce mild clinical symptoms, such as continuous constipation. As a result, the maternal exposure during early pregnancy might be critical for developing congenital aganglionosis or, in milder cases, for the development of less severe motility disorders in children.

One of the main routes of MPs into the body is ingestion. When MPs enter the gastrointestinal tract, they may exert harmful effects through various mechanisms, including microbiota disturbances, mutagenicity, cytotoxicity, reproductive toxicity, neurotoxicity, and increased oxidative stress (Bazeli et al., 2023; Abass et al., 2025).

Exposure to MPs induces gut microbiota dysbiosis, intestinal barrier dysfunction, metabolic perturbations, and neurotoxicity in different rodents and disturbs metabolic dysfunction in the mouse brain and intestine. The results showed that MPs altered microbiota composition, accompanied by metabolic perturbations in the mouse gut and brain. Specifically, *Firmicutes* and *Bacteroidetes* were suggested to be important phyla for MPs exposure, partially dominating further metabolite alterations (Lee et al., 2024). Thus, MPs have been pointed out as disruptors of the gut microbiota, namely, to gastrointestinal dysbiosis (Lu et al., 2019; Li et al., 2020; Lee et al., 2024), which can later cause neurotoxicity and neurodegeneration (Niesler et al., 2021; Singh et al., 2022).

Additional mechanisms by which MPs affect the nervous system are as include inhibition of AChE activity, inflammatory responses, oxidative stress, mitochondrial dysfunction, autophagy–lysosomal injury, and dysbiosis of the intestinal microbiota (Lu et al., 2025).

Regarding specific MPs neurotoxic mechanisms that may negatively affect enteric neuronal growth and migration and thus result in deficient intestinal innervation, we found the following possible explanations: disturbing neuronal generation and synapse formation (Gupta et al., 2024), inhibiting expression of neurodevelopment-related genes, such as brain-derived neurotrophic factor (BDNF) (Lu et al., 2025), reducing neuronal number, inducing abnormal morphology, and affecting neuronal proliferation, differentiation, and migration (Tian et al., 2025).

Confirming our hypothesis, a recent study on pigs demonstrated that oral PET-MPs administration produces histological changes (injury of the apical parts of the villi, accumulations of cellular debris and mucus, eosinophil infiltration, and hyperemia) and also changes in the enteric nervous system of the porcine jejunum (Gałęcka and Całka, 2024).

Further animal experiments showed that when MPs extracted from disposable paper cups were administered to pregnant mice, a moderate exposure equivalent to 3.3 cups daily was sufficient to alter the cecal microbiome, disrupt global metabolic functions, and impair immune health, thereby increasing the risk of neurodegeneration and miscarriage (Chen et al., 2024).

In addition, even low concentrations of PS-NPs in *Caenorhabditis elegans* were sufficient to accumulate in the digestive system, compromise the intestinal barrier and induce a “leaky gut,” and penetrate into extraintestinal tissues. Moreover, NP exposure in *C. elegans* and human cells induced Parkinson’s disease-like symptoms, such as dopaminergic neuronal degeneration, locomotor dysfunction, and accumulation of α-synuclein aggregates (Jeong et al., 2024).

Finally, pregnant rats exposed to polystyrene nanoplastics during gestation and lactation exhibited abnormalities in cortical thickness and neuronal migration, characterized by increased proliferation of cortical cells and a decreased number of deep-layer neurons (Tian et al., 2025).

In summary, exposure to MPs and NPs during early developmental stages may trigger neurotoxicity and inflammatory responses, which could, among other effects, cause abnormal neuronal migration in the gut and impaired intestinal innervation.

## 3 Reducing exposure to microplastics during pregnancy as a preventive measure

Although MPs have reached all ecosystems and we are in a scenario where chronic and daily exposure seems unavoidable, there are ways to reduce it. The timing and duration of exposure during pregnancy play a critical role in the associated risk and harm (Panneerselvam et al., 2025).

We know that there are many compounds, drugs, and hormones that affect the gene-environment during development. The ENS is highly sensitive to these (Heuckeroth and Schäfer, 2016), making it likely that compounds from degrading MPs exert similar effects.

Although it is currently challenging to determine the precise risk without accurate quantification of human exposure, existing evidence from nutritional studies and animal experiments indicates a substantial risk to the fetus. Therefore, we recommend minimizing overall exposure to MPs.

The main sources of MPs exposure during pregnancy are identified as indoor dust (Zhang et al., 2020), tooth paste, food packaging, plastic bottles and cups, biomedical material, and cosmetics (Nair et al., 2025). Of concern here, the use of cosmetics by pregnant women is quite frequent (Li et al., 2019; Chan et al., 2023), maybe due to a lack of risk perception by consumers (Marie et al., 2016). However, we could recently describe the hazard neurotoxic potential of cosmetics for the future born (Jones et al., 2024).

Another important MPs source is dermal exposure through clothing microfibers, which have a non-negligible presence in some textiles, increasing the potential systemic risks. Here, it should be taken into account that common final consumers of textiles with printed PVC are small children, who are the most vulnerable group due to their developmental status (Rovira and Domingo, 2019).

The water intake should also be noted: more than 10^5^ particles have been quantified per liter of bottled water, the majority of which are nanoplastics (Qian et al., 2024). Although both tap and bottled water contain MPs, the concentration is higher in bottled water than in tap water. Taking this evidence into account, pregnant women should be encouraged to drink tap water instead of bottled water to limit the fetal exposure to MPs (Gambino et al., 2022).

The DPCs release millions of MPs when used for hot beverages. Even a moderate consumption of 3.3 cups daily in pregnant mice was sufficient to induce tissue-specific accumulation and metabolic and reproductive toxicities (Chen et al., 2024). The use of tea bags has also been reported to release high quantities of MPs into beverages (Banaei et al., 2024; Yaroslavov et al., 2025; Jayasekara et al., 2026).

Other recommendations for pregnant women (see Table 3) include avoiding single-use plastics and choosing MPs-free personal care products (Panneerselvam et al., 2025), keeping off seafood (Ferrante et al., 2022; Vega-Herrera et al., 2024), reducing the use of cosmetics (Guerranti et al., 2019), and dressing in natural fibers.

**TABLE 3:** Safeguarding recommendations for pregnant women.

Reduce consumption of	Increase consumption of
Synthetic textiles	Natural fiber textiles (i.e.: cotton, wool, and linen)
Plastic bottled water/drinks	Tap water
Cosmetics	MPs-free personal care products
Disposable plastic/paper cups	MPs-free toothpaste
Packaged food	Prebiotics
Single use plastics	Glass or ceramic pots/recipients
Muscles and fish	Daily home ventilation to remove indoor dust

Finally, the use of feeding bottles, pacifiers, and plastic toys may serve as sources of MPs exposure for infants (Liu et al., 2023) and should therefore be avoided.

As a ray of hope, probiotics consumption seems to play a protective role against PS-MPs. Probiotics can re-balance gut dysbiosis and intestinal leakage induced by MPs, reducing inflammatory biomarkers and avoiding unnecessary activation of the immune system. Thus, probiotics may overcome the toxicity of PS-MPs in humans (Bazeli et al., 2023), and its consumption may be recommendable during pregnancy.

## 4 Concluding remarks

Altogether, microplastics may act as neurotoxicants, affect the nervous system, cross the placenta, and reach the fetus, making them likely disruptors of enteric nervous system development and proper intestinal innervation during fetal growth.

Overall, microplastic neurotoxicity appears as an aggravating factor for developing congenital enteric aganglionosis and, therefore, for postnatal motility disorders.

Therefore, although human exposure to microplastics has not yet been adequately measured and the precise risk remains uncertain, we recommend minimizing the exposure of future mothers to microplastics to protect the developing fetus from potential neurotoxic effects.

## References

[B1] Abass D. Alaraby M. Elkady E. F. Morataya-Reyes M. Banaei G. Martín-Pérez J. (2025). Polytetrafluoroethylene (PTFE, Teflon) microplastics and nanoplastics induce oxidative stress, mitochondrial damage, and genotoxicity in human intestinal cells. J. Hazard. Mat. 499, 140255. 10.1016/j.jhazmat.2025.140255 41167152

[B2] Aengenheister L. Keevend K. Muoth C. Schönenberger R. Diener L. Wick P. (2018). An advanced human *in vitro* co-culture model for translocation studies across the placental barrier. Sci. Rep. 8, 5388. 10.1038/s41598-018-23410-6 29599470 PMC5876397

[B3] Altmann K. Wimmer L. Alcolea-Rodriguez V. Waniek T. Wachtendorf V. Matzdorf K. (2025). Quality-by-design and current good practices for the production of test and reference materials for micro- and nano-plastic research. J. Hazard. Mat. 497, 139595. 10.1016/j.jhazmat.2025.139595 40845574

[B4] Baeza-Martínez C. Olmos S. González-Pleiter M. López-Castellanos J. García-Pachón E. Masiá-Canuto M. (2022). First evidence of microplastics isolated in European citizens’ lower airway. J. Hazard. Mat. 438, 129439. 10.1016/j.jhazmat.2022.129439 35777146

[B5] Bai C.-L. Wang D. Luan Y.-L. Huang S.-N. Liu L.-Y. Guo Y. (2024). A review on micro- and nanoplastics in humans: implication for their translocation of barriers and potential health effects. Chemosphere 361, 142424. 10.1016/j.chemosphere.2024.142424 38795915

[B6] Banaei G. Abass D. Tavakolpournegari A. Martín-Pérez J. Gutiérrez J. Peng G. (2024). Teabag-derived micro/nanoplastics (true-to-life MNPLs) as a surrogate for real-life exposure scenarios. Chemosphere 368, 143736. 10.1016/j.chemosphere.2024.143736 39542373

[B7] Bazeli J. Banikazemi Z. Hamblin M. R. Sharafati Chaleshtori R. (2023). Could probiotics protect against human toxicity caused by polystyrene nanoplastics and microplastics? Front. Nutr. 10, 1186724. 10.3389/fnut.2023.1186724 37492595 PMC10363603

[B8] Bhattacharyya S. Greer M. L. Salehi M. (2025). Impact of micro- and nanoplastics exposure on human health: focus on neurological effects from ingestion. Front. Public Health 13, 1681776. 10.3389/fpubh.2025.1681776 41246084 PMC12616133

[B9] Bissonnette J. R. Harvey N. E. Rowsell M. L. Kiefte S. Houthuijs K. J. Béen F. M. (2025). Identification of micro-/nanoplastics in human placental blood using comprehensive multidimensional pyrolysis - gas chromatography x ion mobility mass spectrometry. Anal. Chim. Acta 1376, 344606. 10.1016/j.aca.2025.344606 41073001

[B10] Brachner A. Fragouli D. Duarte I. F. Farias P. M. A. Dembski S. Ghosh M. (2020). Assessment of human health risks posed by nano-and microplastics is currently not feasible. Int. J. Environ. Res. Public Health 17, 8832. 10.3390/ijerph17238832 33261100 PMC7730001

[B11] Braga M. F. M. Pereira E. F. R. Marchioro M. Albuquerque E. X. (1999). Lead increases tetrodotoxin-insensitive spontaneous release of glutamate and GABA from hippocampal neurons. Brain Res. 826, 10–21. 10.1016/S0006-8993(99)01193-2 10216192

[B12] Braun T. Ehrlich L. Henrich W. Koeppel S. Lomako I. Schwabl P. (2021). Detection of microplastic in human placenta and meconium in a clinical setting. Pharmaceutics 13, 921. 10.3390/pharmaceutics13070921 34206212 PMC8308544

[B13] Campanale M. Massarelli C. Savino I. Locaputo V. Uricchio V. F. (2020). A detailed review study on potential effects of microplastics and additives of concern on human health. Int. J. Environ. Res. Public Health 17 (4), 1212. 10.3390/ijerph17041212 32069998 PMC7068600

[B14] Campen M. J. West A. B. Garcia M. Gullapalli R. El Hayek E. (2025). Reply to: challenges in studying microplastics in human brain. Nat. Med. 31, 4036–4037. 10.1038/s41591-025-04046-2 41233598

[B15] Catalán J. Afanou A. K. Arranz J. A. Riaza A. B. Banić I. Dirven H. (2025). An integrated approach to assess exposure and early health effects in human populations exposed to micro- and nanoplastics. NanoImpact 40, 100600. 10.1016/j.impact.2025.100600 41314612

[B16] Chan M. Preston E. V. Fruh V. Quinn M. R. Hacker M. R. Wylie B. J. (2023). Use of personal care products during pregnancy and birth outcomes – a pilot study. Environ. Res. 225, 115583. 10.1016/j.envres.2023.115583 36868449 PMC10153796

[B17] Chen Q. Peng C. Xie R. Xu H. Su Z. Yilihan G. (2024). Placental and fetal enrichment of microplastics from disposable paper cups: implications for metabolic and reproductive health during pregnancy. J. Hazard. Mat. 478, 135527. 10.1016/j.jhazmat.2024.135527 39151363

[B18] Chew K. C. M. Ang E.-T. Tai Y. K. Tsang F. Lo S. Q. Ong E. (2011). Enhanced autophagy from chronic toxicity of iron and mutant A53T α-Synuclein. J. Biol. Chem. 286, 33380–33389. 10.1074/jbc.M111.268409 21795716 PMC3190914

[B19] Cox K. D. Covernton G. A. Davies H. L. Dower J. F. Juanes F. Dudas S. E. (2019). Human consumption of microplastics. Environ. Sci. Technol. 53, 7068–7074. 10.1021/acs.est.9b01517 31184127

[B20] Cubas A. L. V. Bianchet R. T. Reis I. M. A. S. dos Gouveia I. C. (2022). Plastics and microplastic in the cosmetic industry: aggregating sustainable actions aimed at alignment and interaction with UN sustainable development goals. Polym. (Basel) 14, 4576. 10.3390/polym14214576 36365573 PMC9657586

[B21] Deng Y. Zhang Y. Lemos B. Ren H. (2017). Tissue accumulation of microplastics in mice and biomarker responses suggest widespread health risks of exposure. Sci. Rep. 7, 46687. 10.1038/srep46687 28436478 PMC5402289

[B22] DiNardo J. C. Downs C. A. (2019). Can oxybenzone cause Hirschsprung’s disease? Reprod. Toxicol. 86, 98–100. 10.1016/j.reprotox.2019.02.014 30831214

[B23] Dubey I. Khan S. Kushwaha S. (2022). Developmental and reproductive toxic effects of exposure to microplastics: a review of associated signaling pathways. Front. Toxicol. 4, 901798. 10.3389/ftox.2022.901798 36119356 PMC9471315

[B24] Durkin A. M. Zou R. Boucher J. M. Boyles M. S. van Boxel J. Bustamante M. (2024). Investigating exposure and hazards of Micro- and nanoplastics during pregnancy and early life (AURORA project): protocol for an interdisciplinary study. JMIR Res. Protoc. 13, e63176. 10.2196/63176 39378424 PMC11496927

[B25] Evangelou I. Bucci S. Stohl A. (2026). Atmospheric microplastic emissions from land and ocean. Nature 649, 1186–1189. 10.1038/s41586-025-09998-6 41565806 PMC12851922

[B26] Ferrante M. Pietro Z. Allegui C. Maria F. Antonio C. Pulvirenti E. (2022). Microplastics in fillets of Mediterranean seafood. A risk assessment study. Environ. Res. 204, 112247. 10.1016/j.envres.2021.112247 34678256

[B27] Fournier E. Ratel J. Denis S. Leveque M. Ruiz P. Mazal C. (2023). Exposure to polyethylene microplastics alters immature gut microbiome in an infant *in vitro* gut model. J. Hazard. Mat. 443, 130383. 10.1016/j.jhazmat.2022.130383 36444070

[B28] Freire C. Amaya E. Gil F. Fernández M. F. Murcia M. Llop S. (2018). Prenatal co-exposure to neurotoxic metals and neurodevelopment in preschool children: the environment and childhood (INMA) project. Sci. Total Environ. 621, 340–351. 10.1016/j.scitotenv.2017.11.273 29190557

[B29] Freire C. Amaya E. Gil F. Murcia M. LLop S. Casas M. (2019). Placental metal concentrations and birth outcomes: the environment and childhood (INMA) project. Int. J. Hyg. Environ. Health 222, 468–478. 10.1016/j.ijheh.2018.12.014 30638867

[B30] Gałęcka I. Całka J. (2024). Oral exposure to microplastics affects the neurochemical plasticity of reactive neurons in the Porcine Jejunum. Nutrients 16, 2268. 10.3390/nu16142268 39064711 PMC11280339

[B31] Gambino I. Bagordo F. Grassi T. Panico A. De Donno A. (2022). Occurrence of microplastics in tap and bottled water: current knowledge. Int. J. Environ. Res. Public Health 19, 5283. 10.3390/ijerph19095283 35564678 PMC9103198

[B32] Gelbke H.-P. Banton M. Block C. Dawkins G. Eisert R. Leibold E. (2019). Risk assessment for migration of styrene oligomers into food from polystyrene food containers. Food Chem. Toxicol. 124, 151–167. 10.1016/j.fct.2018.11.017 30419324

[B33] Geyer R. Jambeck J. R. Law K. L. (2017). Production, use, and fate of all plastics ever made. Sci. Adv. 3, e1700782. 10.1126/sciadv.1700782 28776036 PMC5517107

[B34] Guerranti C. Martellini T. Perra G. Scopetani C. Cincinelli A. (2019). Microplastics in cosmetics: environmental issues and needs for global bans. Environ. Toxicol. Pharmacol. 68, 75–79. 10.1016/j.etap.2019.03.007 30877953

[B35] Gupta P. Mahapatra A. Manna B. Suman A. Ray S. S. Singhal N. (2024). Sorption of PFOS onto polystyrene microplastics potentiates synergistic toxic effects during zebrafish embryogenesis and neurodevelopment. Chemosphere 366, 143462. 10.1016/j.chemosphere.2024.143462 39368493

[B36] Gutiérrez-García J. Egea R. Barguilla I. Nymark P. García-Rodríguez A. Guyot B. (2025). Long-term exposure to real-life polyethylene terephthalate nanoplastics induces carcinogenesis *in vitro* . Environ. Sci. Technol. 59, 10891–10904. 10.1021/acs.est.5c01628 40452141 PMC12164274

[B37] Heuckeroth R. O. (2015). Hirschsprung’s disease, down syndrome, and missing heritability: too much collagen slows migration. J. Clin. Investigation 125, 4323–4326. 10.1172/JCI85003 26571392 PMC4665790

[B38] Heuckeroth R. O. (2018). Hirschsprung disease—integrating basic science and clinical medicine to improve outcomes. Nat. Rev. Gastroenterol. Hepatol. 15, 152–167. 10.1038/nrgastro.2017.149 29300049

[B39] Heuckeroth R. O. Schäfer K.-H. (2016). Gene-environment interactions and the enteric nervous system: neural plasticity and Hirschsprung disease prevention. Dev. Biol. 417, 188–197. 10.1016/j.ydbio.2016.03.017 26997034 PMC5026873

[B40] Hu J. Qin X. Zhang J. Zhu Y. Zeng W. Lin Y. (2021). Polystyrene microplastics disturb maternal-fetal immune balance and cause reproductive toxicity in pregnant mice. Reprod. Toxicol. 106, 42–50. 10.1016/j.reprotox.2021.10.002 34626775

[B41] Huo W. Cai P. Chen M. Li H. Tang J. Xu C. (2016). The relationship between prenatal exposure to BP-3 and Hirschsprung’s disease. Chemosphere 144, 1091–1097. 10.1016/j.chemosphere.2015.09.019 26454118

[B42] Iribarne-Durán L. M. Peinado F. M. Freire C. Castillero-Rosales I. Artacho-Cordón F. Olea N. (2022). Concentrations of bisphenols, parabens, and benzophenones in human breast milk: a systematic review and meta-analysis. Sci. Total Environ. 806, 150437. 10.1016/j.scitotenv.2021.150437 34583069

[B43] Jayasekara P. M. Abhishek P. Kahandawala B. S. Damith N. Weerasinghe M. Kahatapitiya N. S. (2026). Brewing plastics: OCT reveals microplastic release from nylon tea bags in simulated brewed tea infusions. Environ. Sci. Process. Impacts. 28, 392–404. 10.1039/D5EM00644A 41504735

[B44] Jeong A. Park S. J. Lee E. J. Kim K. W. (2024). Nanoplastics exacerbate Parkinson’s disease symptoms in *C. elegans* and human cells. J. Hazard. Mat. 465, 133289. 10.1016/j.jhazmat.2023.133289 38157817

[B45] Jin Y. Xia J. Pan Z. Yang J. Wang W. Fu Z. (2018). Polystyrene microplastics induce microbiota dysbiosis and inflammation in the gut of adult zebrafish. Environ. Pollut. 235, 322–329. 10.1016/j.envpol.2017.12.088 29304465

[B46] Jin Y. Lu L. Tu W. Luo T. Fu Z. (2019). Impacts of polystyrene microplastic on the gut barrier, microbiota and metabolism of mice. Sci. Total Environ. 649, 308–317. 10.1016/j.scitotenv.2018.08.353 30176444

[B47] Jones K. Wessel L. M. Schäfer K.-H. Tapia-Laliena M. Á. (2024). Use of cosmetics in pregnancy and neurotoxicity: can it increase the risk of congenital enteric neuropathies? Biomolecules 14, 984. 10.3390/biom14080984 39199372 PMC11352589

[B48] Julienne F. Delorme N. Lagarde F. (2019). From macroplastics to microplastics: role of water in the fragmentation of polyethylene. Chemosphere 236, 124409. 10.1016/j.chemosphere.2019.124409 31545205

[B49] Kim J. Chen M. White R. S. (2025). Microplastics and the placenta: a call to action for perinatal research. Am. J. Perinatol. 43, 433–436. 10.1055/a-2657-6249 40675609

[B50] Kole P. J. Löhr A. J. Van Belleghem F. Ragas A. (2017). Wear and tear of tyres: a stealthy source of microplastics in the environment. Int. J. Environ. Res. Public Health 14, 1265. 10.3390/ijerph14101265 29053641 PMC5664766

[B51] Kwiatkowska K. Ormaniec P. (2024). Microbial succession on microplastics in wastewater treatment plants: exploring the complexities of microplastic-microbiome interactions. Microb. Ecol. 87, 105. 10.1007/s00248-024-02422-y 39133233 PMC11319512

[B52] Lake J. I. Heuckeroth R. O. (2013). Enteric nervous system development: migration, differentiation, and disease. Am. J. Physiol.-Gastroint. Liver Physiol. 305, G1–G24. 10.1152/ajpgi.00452.2012 23639815 PMC3725693

[B53] Lee S.-H. Lin W.-Y. Cheng T.-J. (2024). Microbiota-mediated metabolic perturbations in the gut and brain of mice after microplastic exposure. Chemosphere 350, 141026. 10.1016/j.chemosphere.2023.141026 38145850

[B54] Leppert B. Strunz S. Seiwert B. Schlittenbauer L. Schlichting R. Pfeiffer C. (2020). Maternal paraben exposure triggers childhood overweight development. Nat. Commun. 11, 561. 10.1038/s41467-019-14202-1 32047148 PMC7012887

[B55] Li H. Zheng J. Wang H. Huang G. Huang Q. Feng N. (2019). Maternal cosmetics use during pregnancy and risks of adverse outcomes: a prospective cohort study. Sci. Rep. 9, 8030. 10.1038/s41598-019-44546-z 31142815 PMC6541712

[B56] Li B. Ding Y. Cheng X. Sheng D. Xu Z. Rong Q. (2020). Polyethylene microplastics affect the distribution of gut microbiota and inflammation development in mice. Chemosphere 244, 125492. 10.1016/j.chemosphere.2019.125492 31809927

[B57] Liu S. Guo J. Liu X. Yang R. Wang H. Sun Y. (2023). Detection of various microplastics in placentas, meconium, infant feces, breastmilk and infant formula: a pilot prospective study. Sci. Total Environ. 854, 158699. 10.1016/j.scitotenv.2022.158699 36108868

[B58] Loff S. Waag K.-L. Kränzlin B. Zovko D. Dzakovic A. Jester I. (1998). Long-term total parenteral nutrition-induced hepatobiliary dysfunction in a rabbit model. J. Pediatr. Surg. 33, 694–699. 10.1016/S0022-3468(98)90189-0 9607470

[B59] Loff S. Kränzlin B. Moghadam M. Dzakovic A. Wessel L. Back W. (1999). Parenteral nutrition-induced hepatobiliary dysfunction in infants and prepubertal rabbits. Pediatr. Surg. Int. 15, 479–482. 10.1007/s003830050643 10525903

[B60] Loff S. Kabs F. Witt K. Sartoris J. Mandl B. Niessen K. H. (2000). Polyvinylchloride infusion lines expose infants to large amounts of toxic plasticizers. J. Pediatr. Surg. 35, 1775–1781. 10.1053/jpsu.2000.19249 11101735

[B61] Lu L. Luo T. Zhao Y. Cai C. Fu Z. Jin Y. (2019). Interaction between microplastics and microorganism as well as gut microbiota: a consideration on environmental animal and human health. Sci. Total Environ. 667, 94–100. 10.1016/j.scitotenv.2019.02.380 30826685

[B62] Lu K. Que Y. Wang L. Wang Y. Qiu J. Jia Y. (2025). Environmental exposure pathways of microplastics and their toxic effects on ecosystems and the nervous system. Front. Toxicol. 7, 1649282. 10.3389/ftox.2025.1649282 41048613 PMC12491214

[B63] Luo T. Zhang Y. Wang C. Wang X. Zhou J. Shen M. (2019). Maternal exposure to different sizes of polystyrene microplastics during gestation causes metabolic disorders in their offspring. Environ. Pollut. 255, 113122. 10.1016/j.envpol.2019.113122 31520900

[B64] Luzón‐Toro B. Villalba‐Benito L. Torroglosa A. Fernández R. M. Antiñolo G. Borrego S. (2020). What is new about the genetic background of Hirschsprung disease? Clin. Genet. 97, 114–124. 10.1111/cge.13615 31355911

[B65] Marie C. Cabut S. Vendittelli F. Sauvant-Rochat M.-P. (2016). Changes in cosmetics use during pregnancy and risk perception by women. Int. J. Environ. Res. Public Health 13, 383. 10.3390/ijerph13040383 27043593 PMC4847045

[B66] Mejías C. Martín J. Santos J. L. Aparicio I. Alonso E. (2023). Role of polyamide microplastics as vector of parabens in the environment: an adsorption study. Environ. Technol. Innov. 32, 103276. 10.1016/j.eti.2023.103276

[B67] Menezes M. Puri P. (2006). Long-term outcome of patients with enterocolitis complicating Hirschsprung’s disease. Pediatr. Surg. Int. 22, 316–318. 10.1007/s00383-006-1639-2 16463033

[B68] Meyrueix L. Adair L. Norris S. A. Ideraabdullah F. (2019). Assessment of placental metal levels in a South African cohort. Environ. Monit. Assess. 191, 500. 10.1007/s10661-019-7638-2 31321551 PMC6681656

[B69] Monikh A. Materić D. Valsami-Jones E. Grossart H.-P. Altmann K. Holzinger R. (2025). Challenges in studying microplastics in human brain. Nat. Med. 31, 4034–4035. 10.1038/s41591-025-04045-3 41233597

[B70] Montano L. Raimondo S. Piscopo M. Ricciardi M. Guglielmino A. Chamayou S. (2025). First evidence of microplastics in human ovarian follicular fluid: an emerging threat to female fertility. Ecotoxicol. Environ. Saf. 291, 117868. 10.1016/j.ecoenv.2025.117868 39947063

[B71] Nair S. R. Nihad M. Shenoy P S. Gupta S. Bose B. (2025). Unveiling the effects of micro and nano plastics in embryonic development. Toxicol. Rep. 14, 101954. 10.1016/j.toxrep.2025.101954 40104046 PMC11914762

[B72] Nardella F. Brits M. van Velzen M. J. M. Scibetta L. Durkin A. Vermeulen R. (2025). Advancing pyrolysis-gas chromatography-mass spectrometry for the accurate quantification of micro- and nanoplastics in human blood. Micropl. Nanoplast. 5, 48. 10.1186/s43591-025-00152-7 41459190 PMC12738653

[B73] Niesler B. Kuerten S. Demir I. E. Schäfer K.-H. (2021). Disorders of the enteric nervous system — a holistic view. Nat. Rev. Gastroenterol. Hepatol. 18, 393–410. 10.1038/s41575-020-00385-2 33514916

[B74] Nihart A. J. Garcia M. A. El Hayek E. Liu R. Olewine M. Kingston J. D. (2025). Bioaccumulation of microplastics in decedent human brains. Nat. Med. 31, 1114–1119. 10.1038/s41591-024-03453-1 39901044 PMC12003191

[B75] Oliveri Conti G. Ferrante M. Banni M. Favara C. Nicolosi I. Cristaldi A. (2020). Micro- and nano-plastics in edible fruit and vegetables. The first diet risks assessment for the general population. Environ. Res. 187, 109677. 10.1016/j.envres.2020.109677 32454310

[B76] Pahwa R. Kalra J. (1993). A critical review of the neurotoxicity of styrene in humans. Vet. Hum. Toxicol. 35 (6), 516–520. 7980742

[B77] Panneerselvam D. Murugesan A. Raveendran S. K. Kumar J. S. Venkataraman P. (2025). Examining the hidden dangers: understanding how microplastics affect pregnancy. Eur. J. Obstet. and Gynecol. Reprod. Biol. 304, 53–62. 10.1016/j.ejogrb.2024.11.024 39580908

[B78] Paruta P. Pucino M. Boucher J. (2022). Plastic paints the environment. Lausanne, Switzerland: EA – Environmental Action.

[B79] Pohanka M. (2014). Copper, aluminum, iron and calcium inhibit human acetylcholinesterase *in vitro* . Environ. Toxicol. Pharmacol. 37, 455–459. 10.1016/j.etap.2014.01.001 24473150

[B80] Prüst M. Meijer J. Westerink R. H. S. (2020). The plastic brain: neurotoxicity of micro- and nanoplastics. Part. Fibre Toxicol. 17, 24. 10.1186/s12989-020-00358-y 32513186 PMC7282048

[B81] Qian N. Gao X. Lang X. Deng H. Bratu T. M. Chen Q. (2024). Rapid single-particle chemical imaging of nanoplastics by SRS microscopy. Proc. Natl. Acad. Sci. 121, e2300582121. 10.1073/pnas.2300582121 38190543 PMC10801917

[B82] Qu M. Wang D. (2020). Toxicity comparison between pristine and sulfonate modified nanopolystyrene particles in affecting locomotion behavior, sensory perception, and neuronal development in *Caenorhabditis elegans* . Sci. Total Environ. 703, 134817. 10.1016/j.scitotenv.2019.134817 31715464

[B83] Rafa N. Ahmed B. Zohora F. Bakya J. Ahmed S. Ahmed S. F. (2024). Microplastics as carriers of toxic pollutants: source, transport, and toxicological effects. Environ. Pollut. 343, 123190. 10.1016/j.envpol.2023.123190 38142809

[B84] Ragusa A. Svelato A. Santacroce C. Catalano P. Notarstefano V. Carnevali O. (2021). Plasticenta: first evidence of microplastics in human placenta. Environ. Int. 146, 106274. 10.1016/j.envint.2020.106274 33395930

[B85] Rodier P. M. (1995). Developing brain as a target of toxicity. Environ. Health Perspect. 103, 73–76. 10.1289/ehp.95103s673 8549496 PMC1518932

[B86] Rovira J. Domingo J. L. (2019). Human health risks due to exposure to inorganic and organic chemicals from textiles: a review. Environ. Res. 168, 62–69. 10.1016/j.envres.2018.09.027 30278363

[B87] Saraluck A. Techarang T. Bunyapipat P. Boonchuwong K. Pullaput Y. Mordmuang A. (2024). Detection of microplastics in human breast milk and its association with changes in human milk bacterial microbiota. J. Clin. Med. 13, 4029. 10.3390/jcm13144029 39064070 PMC11277308

[B88] Schwabl P. Köppel S. Königshofer P. Bucsics T. Trauner M. Reiberger T. (2019). Detection of various microplastics in human stool. Ann. Intern. Med. 171, 453–457. 10.7326/M19-0618 31476765

[B89] Senathirajah K. Attwood S. Bhagwat G. Carbery M. Wilson S. Palanisami T. (2021). Estimation of the mass of microplastics ingested – a pivotal first step towards human health risk assessment. J. Hazard. Mat. 404, 124004. 10.1016/j.jhazmat.2020.124004 33130380

[B90] Serafim A. Company R. Lopes B. Rosa J. Cavaco A. Castela G. (2012). Assessment of essential and nonessential metals and different metal exposure biomarkers in the human placenta in a population from the south of Portugal. J. Toxicol. Environ. Health A 75, 867–877. 10.1080/15287394.2012.690704 22788373

[B91] Singh S. Sharma P. Pal N. Kumawat M. Shubham S. Sarma D. K. (2022). Impact of environmental pollutants on gut microbiome and mental health *via* the gut–brain axis. Microorganisms 10, 1457. 10.3390/microorganisms10071457 35889175 PMC9317668

[B92] Sökmen T. Ö. Sulukan E. Türkoğlu M. Baran A. Özkaraca M. Ceyhun S. B. (2020). Polystyrene nanoplastics (20 nm) are able to bioaccumulate and cause oxidative DNA damages in the brain tissue of zebrafish embryo (*Danio rerio*). Neurotoxicology 77, 51–59. 10.1016/j.neuro.2019.12.010 31862285

[B93] Sun Q. Ren S.-Y. Ni H.-G. (2020). Incidence of microplastics in personal care products: an appreciable part of plastic pollution. Sci. Total Environ. 742, 140218. 10.1016/j.scitotenv.2020.140218 32629242

[B94] Sun H. Su X. Mao J. Liu Y. Li G. Du Q. (2024). Microplastics in maternal blood, fetal appendages, and umbilical vein blood. Ecotoxicol. Environ. Saf. 287, 117300. 10.1016/j.ecoenv.2024.117300 39509785

[B95] Tang C. S. Li P. Lai F. P.-L. Fu A. X. Lau S.-T. So M. T. (2018). Identification of genes associated with Hirschsprung disease, based on whole-genome sequence analysis, and potential effects on enteric nervous system development. Gastroenterology 155, 1908–1922.e5. 10.1053/j.gastro.2018.09.012 30217742

[B96] Thompson R. C. Courtene-Jones W. Boucher J. Pahl S. Raubenheimer K. Koelmans A. A. (2024). Twenty years of microplastic pollution research—what have we learned? Science 386 (1979), 386. 10.1126/science.adl2746 39298564

[B97] Tian L. Chen J. Liu X. Wei Y. Zhao Y. Shi Y. (2025). Prenatal exposure on nanoplastics: a study of spatial transcriptomics in hippocampal offspring. Environ. Pollut. 366, 125480. 10.1016/j.envpol.2024.125480 39644950

[B98] Tseng I.-L. Yang Y.-F. Yu C.-W. Li W.-H. Liao V. H.-C. (2013). Phthalates induce neurotoxicity affecting locomotor and thermotactic behaviors and AFD neurons through oxidative stress in *Caenorhabditis elegans* . PLoS One 8, e82657. 10.1371/journal.pone.0082657 24349328 PMC3861438

[B99] Ullah S. Ahmad S. Guo X. Ullah S. Ullah S. Nabi G. (2023). A review of the endocrine disrupting effects of micro and nano plastic and their associated chemicals in mammals. Front. Endocrinol. (Lausanne) 13, 1084236. 10.3389/fendo.2022.1084236 36726457 PMC9885170

[B100] van Raamsdonk L. W. D. van der Zande M. Koelmans A. A. Hoogenboom R. L. A. P. Peters R. J. B. Groot M. J. (2020). Current insights into monitoring, bioaccumulation, and potential health effects of microplastics present in the food chain. Foods 9, 72. 10.3390/foods9010072 31936455 PMC7022559

[B101] Vega-Herrera A. Savva K. Lacoma P. Santos L. H. M. L. M. Hernández A. Marmelo I. (2024). Bioaccumulation and dietary bioaccessibility of microplastics composition and cocontaminants in Mediterranean mussels. Chemosphere 363, 142934. 10.1016/j.chemosphere.2024.142934 39053781

[B102] Vela-Soria F. Gallardo-Torres M. E. Ballesteros O. Díaz C. Pérez J. Navalón A. (2017). Assessment of parabens and ultraviolet filters in human placenta tissue by ultrasound-assisted extraction and ultra-high performance liquid chromatography-tandem mass spectrometry. J. Chromatogr. A 1487, 153–161. 10.1016/j.chroma.2017.01.041 28129936

[B103] Vercauteren M. Panneel L. Jorens P. G. Covaci A. Cleys P. Mulder A. (2024). An *ex vivo* study examining migration of microplastics from an infused neonatal parenteral nutrition circuit. Environ. Health Perspect. 132, 37703. 10.1289/EHP13491 38506503 PMC10953496

[B104] Weis J. S. Alava J. J. (2023). (Micro)Plastics are toxic pollutants. Toxics 11, 935. 10.3390/toxics11110935 37999586 PMC10675727

[B105] Wójtowicz A. K. Szychowski K. A. Wnuk A. Kajta M. (2017). Dibutyl phthalate (DBP)-induced apoptosis and neurotoxicity are mediated *via* the aryl hydrocarbon receptor (AhR) but not by estrogen receptor alpha (ERα), estrogen receptor beta (ERβ), or peroxisome proliferator-activated receptor gamma (PPARγ) in mouse cortical neurons. Neurotox. Res. 31, 77–89. 10.1007/s12640-016-9665-x 27581038 PMC5209414

[B106] Yaroslavov A. A. Efimova A. A. Grokhovskaya T. E. Badikova A. G. Spiridonov V. V. Pozdyshev D. V. (2025). Evolution of microplastics released from tea bags into water. Polym. (Basel) 17, 2700. 10.3390/polym17192700 41096345 PMC12526631

[B107] Zhang J. Cao R. Cai T. Aschner M. Zhao F. Yao T. (2013). The role of autophagy dysregulation in manganese-induced dopaminergic neurodegeneration. Neurotox. Res. 24, 478–490. 10.1007/s12640-013-9392-5 23604964 PMC3785712

[B108] Zhang J. Wang L. Kannan K. (2020). Microplastics in house dust from 12 countries and associated human exposure. Environ. Int. 134, 105314. 10.1016/j.envint.2019.105314 31756678

[B109] Zhang J. Wang L. Trasande L. Kannan K. (2021). Occurrence of polyethylene terephthalate and polycarbonate microplastics in infant and adult feces. Environ. Sci. Technol. Lett. 8, 989–994. 10.1021/acs.estlett.1c00559

[B110] Zhang D. Xiao J. Xiao Q. Chen Y. Li X. Zheng Q. (2023). Infant exposure to parabens, triclosan, and triclocarban *via* breastfeeding and formula supplementing in southern China. Sci. Total Environ. 858, 159820. 10.1016/j.scitotenv.2022.159820 36349623

[B111] Zheng Y. Xu S. Liu J. Liu Z. (2024). The effects of micro- and nanoplastics on the central nervous system: a new threat to humanity? Toxicology 504, 153799. 10.1016/j.tox.2024.153799 38608860

[B112] Zhu L. Kang Y. Ma M. Wu Z. Zhang L. Hu R. (2024). Tissue accumulation of microplastics and potential health risks in human. Sci. Total Environ. 915, 170004. 10.1016/j.scitotenv.2024.170004 38220018

